# Optimal Perioperative Fluid Therapy Associates with Fewer Complications After Pancreaticoduodenectomy

**DOI:** 10.1007/s11605-022-05453-3

**Published:** 2022-09-21

**Authors:** Piia Peltoniemi, Pertti Pere, Harri Mustonen, Hanna Seppänen

**Affiliations:** 1 Department of Perioperative, Intensive Care and Pain Medicine, Faculty of Medicine, University of Helsinki and Helsinki University Hospital, Helsinki, Finland; 2 Department of Gastroenterological Surgery, Faculty of Medicine, University of Helsinki and Helsinki University Hospital, Helsinki, Finland; 3 Translational Cancer Medicine Research Program, Faculty of Medicine, University of Helsinki and Helsinki University Hospital, Helsinki, Finland

**Keywords:** Pancreaticoduodenectomy, Goal-directed fluid therapy, Postoperative complications

## Abstract

**Background:**

Optimal fluid management in pancreaticoduodenectomy patients remains contested. We aimed to examine the association between perioperative fluid administration and postoperative complications.

**Methods:**

We studied 168 pancreaticoduodenectomy patients operated in 2015 (*n* = 93) or 2017 (*n* = 75) at Helsinki University Hospital. In 2015, patients received intraoperative fluids following a goal-directed approach and, in 2017, according to anesthesiologist’s clinical practice (conventional fluid management). We analyzed the differences in perioperative fluid administration between the groups, specifically examining the occurrence of severe complications (Clavien–Dindo ≥ III), pancreatic fistulas, cardiovascular complications, and the length of hospital stay.

**Results:**

The goal-directed group received more intraoperative fluids than the conventional fluid management group (12.0 ml/kg/h vs. 8.3 ml/kg/h, *p* < 0.001). Urine output (770 ml vs. 575 ml, *p* = 0.004) and intraoperative fluid balance (9.4 ml/kg/h vs. 6.3 ml/kg/h, *p* < 0.001) were higher in the goal-directed group than in the conventional fluid management group. Severe surgical complications (19.4% vs. 38.7%, *p* = 0.009) as well as clinically relevant pancreatic fistulas (1.1% vs. 10.7%, *p* = 0.011) occurred more frequently in patients receiving conventional fluid management. Moreover, the conventional fluid management group experienced longer hospital stays (9.0 vs. 11.5 days, *p* = 0.02). Lower intraoperative fluid volume accompanying conventional fluid management was associated with a higher risk of severe postoperative complications compared with higher volume in the goal-directed group (odds ratio 2.58 (95% confidence interval 1.04–6.42), *p* = 0.041).

**Conclusions:**

The goal-directed group experienced severe complications less frequently. Our findings indicate that optimizing the intraoperative fluid administration benefits patients, while adopting a too-restrictive approach represents an inferior choice.

## Introduction

Pancreaticoduodenectomy (PD) is a complex and challenging abdominal surgical procedure. Despite the significant development of surgical techniques in recent decades, complication rates remain high. The most common complications include pancreatic fistulas (POPFs), postoperative hemorrhage, delayed gastric emptying (DGE), and wound infections.^1,2^ Approximately 20–30% of all patients experience severe (Clavien–Dindo ≥ III) complications.^3,4^

One means of reducing the complication rates lies in optimizing patients’ perioperative care and maintaining a normal physiological condition. Specifically designated for this purpose, the enhanced recovery after surgery (ERAS) protocol includes optimal perioperative fluid therapy, effective pain control, an early oral diet, and mobilization. The ERAS protocol reduces morbidity and the length of hospital stay in all abdominal surgery patients^5,6^ and leads to lower DGE rates in PD patients.^7^ Furthermore, ERAS reduces mild postoperative complications (Clavien–Dindo I–II), despite a large meta-analysis finding no impact on severe complications (Clavien–Dindo III–V) or mortality.^8^

Fluid management represents a significant component of the ERAS protocol. The protocol recommends using goal-directed fluid therapy (GDFT) to optimize intraoperative fluid management in high-risk abdominal surgery patients.^9^ GDFT was developed to maintain patient hemodynamics during surgery by dosing catecholamines and fluids in a controlled manner according to specific hemodynamic goals. Thus, the end organs receive sufficient oxygen without delivering excessive fluids. This optimization reduces postoperative complications, intensive care unit (ICU) stays, and hospital stays across all high-risk surgery patients, including patients with a limited respiratory or cardiovascular reserve.^10–12^

The anesthesiologist aims to optimize patient fluid administration during surgery. However, optimal intraoperative fluid therapy in PD patients remains a contested issue with inconsistent findings on the optimal volume of fluids. Administering liberal intraoperative fluids may be associated with more severe complications (Clavien–Dindo ≥ III) and pancreatic fistulas.^13–18^ However, a too-restrictive intraoperative fluid management may increase the occurrence of postoperative pancreatitis and pancreatic fistulas in patients with a soft pancreatic texture.^13,19,20^ In multiple studies, intraoperative fluid administration did not associate with postoperative complication rates in pancreatic surgery patients.^21–24^

Fluid management of pancreatic surgery patients is a topic of interest, whereby the means of administering optimal fluid therapy remains contested. Furthermore, clinical practices vary between anesthesiologists. Thus, our study aimed to (1) clarify whether intraoperative fluid therapy is associated with postoperative complication rates in patients undergoing pancreaticoduodenectomy; (2) assess whether the GDFT method affects the volume of intraoperative fluids required and the postoperative fluid therapy; and (3) evaluate whether postoperative fluid management is associated with complications. We hypothesized that GDFT leads to administering fewer fluids intraoperatively, which, in turn, associates with fewer postoperative complications.

## Materials and Methods

This retrospective cohort study examined patients who underwent PD in 2015 or 2017. Patients underwent surgery at Helsinki University Hospital, a tertiary center for abdominal surgery. The study includes all adult PD patients. We excluded other pancreatic procedures such as total and distal pancreatectomies. In 2015, all PD surgeries were performed at the Surgical Hospital, a separate unit of Helsinki University Hospital with expertise in abdominal surgery. In 2016, all pancreatic operations were transferred to Meilahti Hospital, and all PD patients were operated on there in 2017. Surgeons remained the same at both hospitals along with the majority of anesthesiologists and other staff. In 2017, a somewhat greater number of anesthesiologists and nurses were responsible for patients because the department grew. The institutional review board of the Abdominal Center of Helsinki University Hospital approved the study protocol on 23 March 2020. Due to the retrospective design, ethics committee approval was not needed. The study was registered with ClinicalTrials.gov on 20 December 2020.

Patients operated in 2015 received intraoperative fluids according to a goal-directed strategy. In 2017, patients received intraoperative fluid management according to each anesthesiologist’s clinical practice. Patients who underwent PD in 2016 were excluded from the study because of the transition to a new unit: we preferred choosing two different years consisting of aligned and consistent clinical management during the entire years analyzed, that is, in 2015 and 2017, respectively. We examined the occurrence of severe postoperative complications, pancreatic fistulas, cardiovascular and pulmonary complications, and the length of hospital stays.

**Fig. 1:**
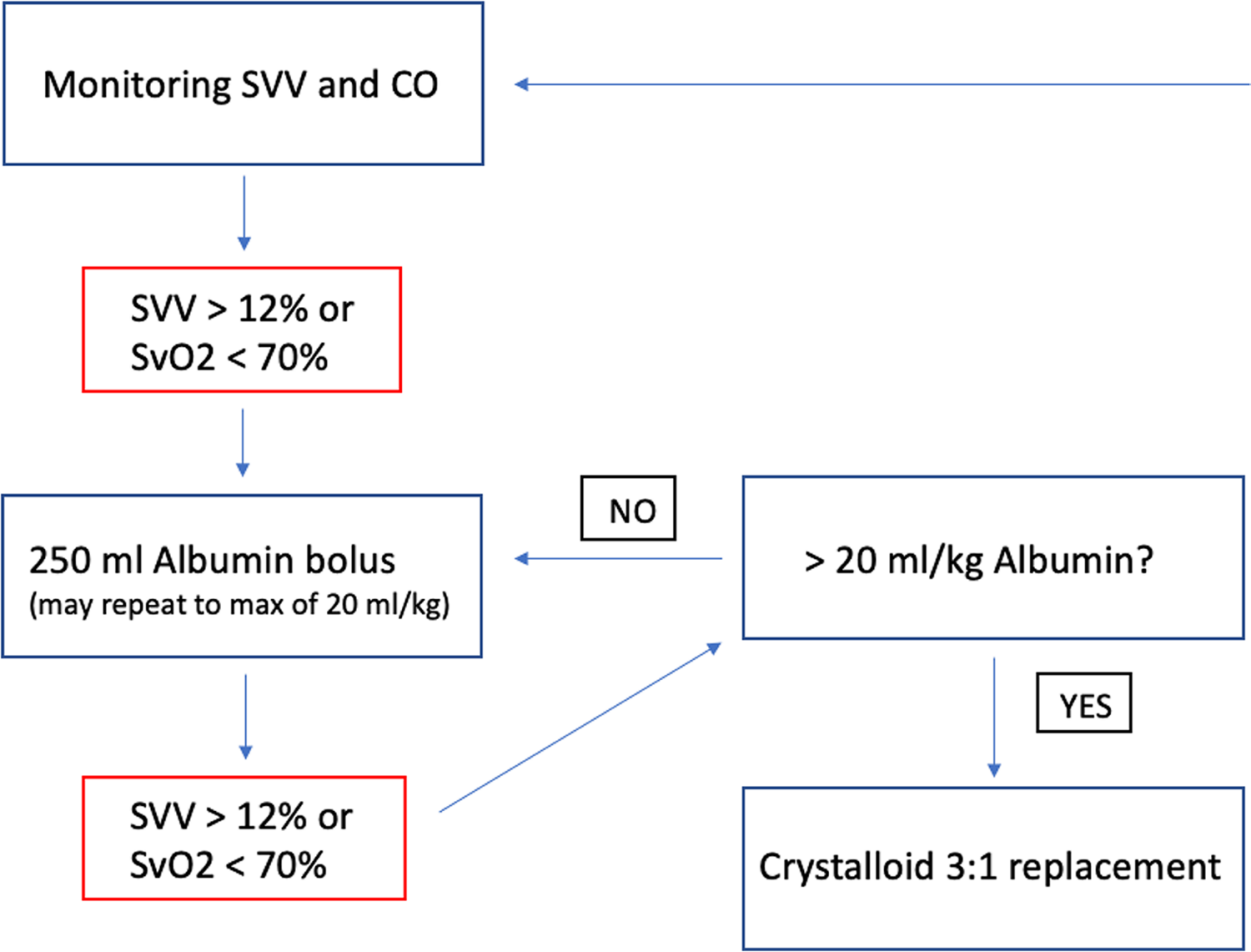
Goal-directed fluid therapy protocol. Modified from the Ramsingh Protocol ^25^. Albumin measured at 40 g/l. SVV, stroke volume variation; SvO2, central venous oxygen saturation

All procedures were performed under general anesthesia and combined with postoperative epidural anesthesia in the absence of contraindications. All patients had an arterial cannula and a central venous catheter. General anesthesia was maintained with an inhalational agent and with fentanyl. In 2015, the arterial cannula and central venous catheter were connected to the FloTrac sensor and the EV1000 monitor (Edwards Lifesciences, Irvine, CA, USA). That system calculated the hemodynamic parameters, such as stroke volume variation (SVV), cardiac index, and cardiac output, according to which the anesthesiologist administered fluids to the patient. A fluid bolus was recommended whenever SVV rose to > 12% or the oxygen hemoglobin saturation in the central venous blood fell to < 70%. Both crystalloids (Ringer-Acetat) and albumin (40 g/l) were administered. The goal-directed protocol employed (Fig. 1) was modified from the Ramsingh Protocol^25^ as it was comprehensive and could be used by all anesthetic staff of the department without device-specific training. In 2017, intraoperative fluids were administered conventionally with no goal-directed strategy based on the consideration of the anesthesiologist. Mean arterial pressure was maintained at > 65 mmHg in all patients with a norepinephrine infusion or the administration of fluids according to the preference of the anesthesiologist.

Perioperative data were retrieved from the institutional electronic database. The intraoperative fluids, blood products, urine output, and overall fluid balance were calculated, as well as the use of norepinephrine. The intraoperative fluid volume was determined including crystalloids, colloids, and infusions of drugs and antibiotics. Similarly, the overall fluid balance was calculated, including the total fluid volume, any blood products, urine output, blood loss, and fluid evaporation. We reviewed all operative reports, but the consistency of the pancreas was defined only in a minority of patients. Thus, we could not analyze different pancreatic textures separately. However, we measured the main pancreatic duct (MPD) diameter on preoperative CT scans and included this to our analyses.

Postoperative care followed the ERAS protocol in both groups. We assessed the fluid management, urine output, and fluid balance for the first four postoperative days (PODs 1–4). In addition, we noted whether the patient was postoperatively admitted to ICU or a regular postoperative ward and whether that influenced fluid management. At the Surgical Hospital in 2015, PD patients were treated in ICU during the first postoperative night whenever possible, while, at Meilahti Hospital in 2017, only patients with significant comorbidities or remarkable intraoperative blood loss were admitted to ICU. Preoperative and postoperative laboratory parameters were reviewed for the first four PODs and radiological imaging for 90 days postoperatively. Drain amylase was measured on POD 3 and subsequently every other day when needed. Drains were removed when the drain amylase fell to < 200 U/l.

Postoperative complications were reported according to the Clavien–Dindo classification.^1^ We regarded Clavien–Dindo I–II as mild complications and Clavien–Dindo III–V as severe complications. We defined surgical complications as severe if they required invasive intervention (i.e., radiological punctures, endoscopy, or re-operation). We determined clinically relevant postoperative pancreatic fistula (grades B–C),^26^ severe post-pancreatectomy hemorrhage (PPH, grades B–C),^27^ severe delayed gastric emptying (grades B–C),^28^ severe post-pancreatectomy acute pancreatitis (PPAP, grades B–C),^29^ and a chyle leak^30^ according to the International Study Group of Pancreatic Surgery (ISGPS) definitions. Congestive heart failure with desaturation and congestion with pleural effusion in radiological imaging were regarded as cardiac complications. We also reported any pleural effusion separately when a pleural puncture was needed. The follow-up time for complications was 90 days postoperatively.

Statistical analyses were performed using IBM® SPSS® Statistics version 26 (IBM, Inc., Armonk, NY, USA). Dichotomized and categorical variables were expressed in numerals and percentages and we used the Fischer’s exact test or the Fisher-Freeman-Halton test in analyses. Continuous variables were expressed as means (standard deviation, SD) or medians (interquartile range, IQR or range) where appropriate’. The Mann–Whitney U test was used for statistical comparisons, while the Shapiro Wilk’s test was used to determine if continuous variables deviated from a normal distribution. We used logistic regression to assess uni- and multivariable analyses for associations between postoperative complications and independent variables. All statistical tests were two-sided, and we considered *p* < 0.05 statistically significant.

## Results

In 2015 and 2017, 288 patients underwent pancreatic surgery at Helsinki University Hospital, with 168 patients undergoing PD. In 2015, 93 PD patients received intraoperative fluid therapy through a goal-directed strategy (GDFT), whereas, in 2017, surgery was performed on 75 PD patients who received conventional fluid management (CFM). Table 1 summarizes the patient characteristics. Pancreatic adenocarcinoma was the most common diagnosis in both groups. Diabetes mellitus was more common in the GDFT group while no other significant differences between groups emerged. Patients with a glomerular filtration rate (GFR) < 60 were regarded as experiencing renal insufficiency.

**Table 1:** Patient characteristics

	GDFT group (*n* = 93)	CFM group (*n* = 75)	*p* value
Age (in years)	69.3 (37.3–81.8)	66.0 (34.8–83.0)*	0.06
Sex			0.35
Male	49 (52.7%)	45 (60.0%)	
Female	44 (47.3%)	30 (40.0%)	
BMI (kg/m^2^)	25.5 (4.4)	26.2 (3.5)**	0.18
ASA classification			0.39
1	3 (3.2%)	3 (4.0%)	
2	27 (29.0%)	26 (34.7%)	
3	57 (61.3%)	45 (60.0%)	
4	6 (6.5%)	1 (1.3%)	
Diagnosis			0.78
Pancreatic adenocarcinoma	57 (61.3%)	40 (54.1%)	
Cholangiocarcinoma	10 (10.8%)	5 (6.8%)	
Papilla vater adenocarcinoma	9 (9.7%)	9 (12.2%)	
Familial adenomatotic polyposis	3 (3.2%)	4 (5.4%)	
MPD ≤ 3 mm	43 (46.2%)	40 (53.3%)	0.61
Preoperative renal function			
Serum creatinine (µmol/l)	69.0 (59.5–84.0)	71.0 (60.0–86.0)	0.82
Renal insufficiency	11 (11.8%)	6 (8.0%)	0.45
High blood pressure	37 (39.8%)	27 (36.0%)	0.64
Diabetes mellitus	28 (30.1%)	12 (16.0%)	*0.04*
Smoking	12 (12.9%)	9 (12.0%)	1.00

^*^Age data are presented as median (range)

^**^BMI data are presented as mean (standard deviation, SD)

Other data are presented as median (interquartile range, IQR) or number (percentage). *GDFT* goal-directed fluid therapy, *CFM* conventional fluid management, *BMI* body mass index, *ASA* American Society of Anesthesiologists, *MPD* main pancreatic duct

**Fig. 2:**
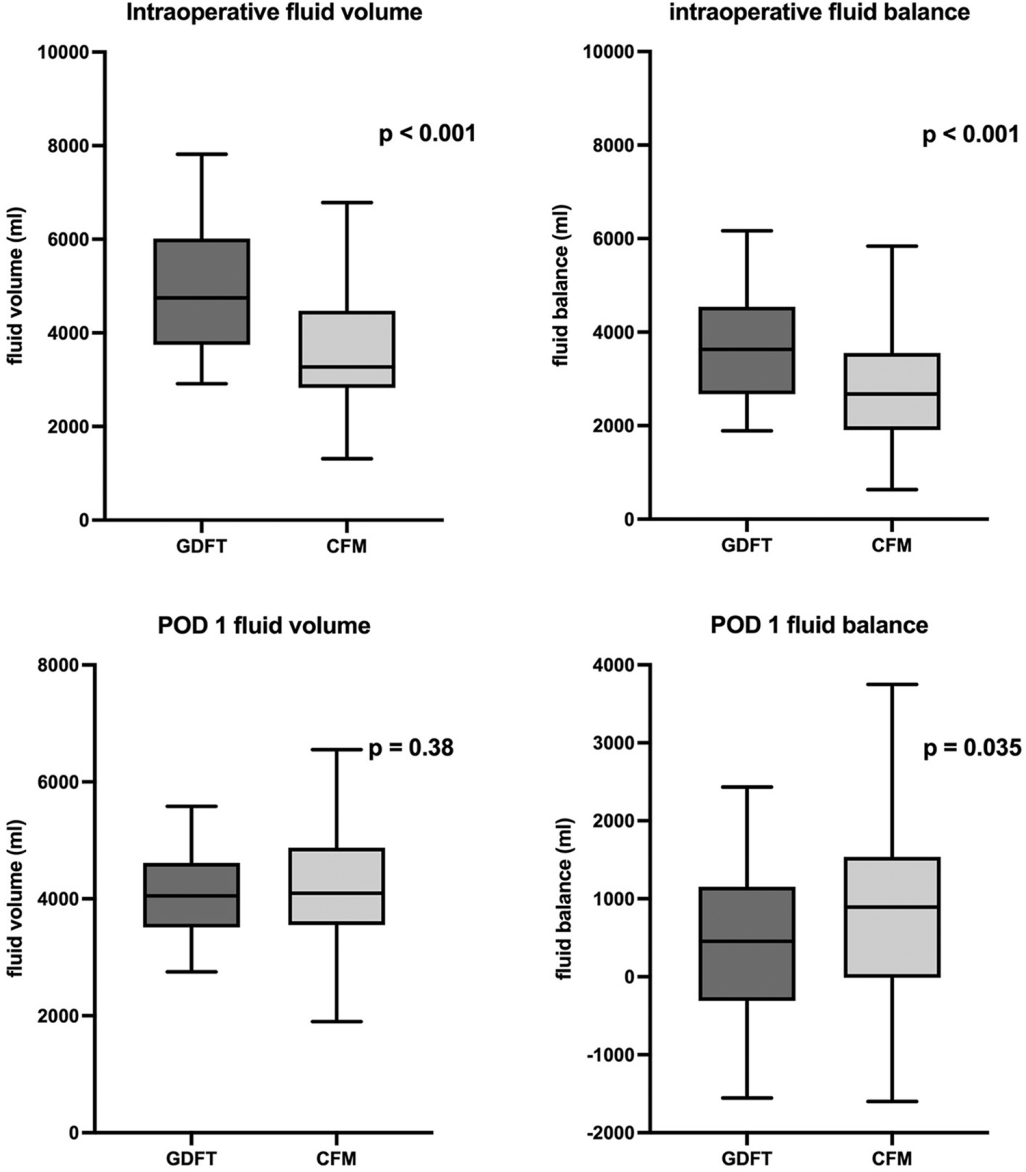
Intraoperative fluid volume and balance, and POD 1 fluid volume and balance in the GDFT and CFM patient groups. Boxes show the median and the interquartile range and the whiskers indicate the 5–95 percentiles. The Mann–Whitney U test was used in the statistical analyses. GDFT, goal-directed fluid therapy; CFM, conventional fluid management; POD, postoperative day

Intraoperatively, patients in the GDFT group received more fluids than those in the CFM group (4750 ml vs. 3271 ml, *p* < 0.001, Fig. 2), which was also the case for intraoperative fluids calculated based on the rate per hour of surgery and patient body weight (12 ml/kg/h vs. 8.3 ml/kg/h, *p* < 0.001). Patients in the CFM group were administered a greater proportion of albumin with respect to the total fluid volume. We identified no differences in vasopressor use. Intraoperative urine output and the intraoperative fluid balance were higher in the GDFT group. We detected no difference between groups in the duration of surgery or the need for blood vessel reconstruction (see Table 2).

**Table 2:** Intraoperative and postoperative fluid management

	GDFT group	CFM group	*p* value
*Intraoperative period*			
Total fluid volume (ml)	4750 (3744–6013)	3271 (2828–4471)	< *0.001*
Total fluid volume (ml/kg/h)	12.01 (9.81–14.25)	8.27 (6.77–9.87)	< *0.001*
Crystalloids (ml)	4000 (3193–5198)	2851 (2215–3715)	< *0.001*
Albumin (g)	32.0 (16.0–48.0)	36.0 (16.0–68.0)	0.56
Albumin/total fluid volume (g/l)	5.81 (3.77–8.48)	8.81 (4.09–16.60)	*0.013*
Red blood cell transfusion, n (%)	42 (47.2%)	17 (23.0%)	*0.002*
Blood loss (ml)	820 (500–1240)	700 (400–1000)	0.053
Urine output (ml)	770 (473–1465)	575 (428–830)	*0.004*
Fluid balance (ml)	3633 (2675–4544)	2678 (1907–3553)	< *0.001*
Norepinephrine infused (mg)	2.13 (1.10–2.92)	2.08 (1.07–3.97)	0.44
Duration of surgery (minutes)	318 (290–383)	318 (265–372)	0.49
Blood vessel reconstruction, n (%)	27 (29.0%)	19 (25.3%)	0.61
Epidural analgesia, n (%)	90 (96.8%)	73 (97.3%)	1.00
*Postoperative period*			
POD 1			
Fluid administered (ml)	4050 (3513–4615)	4097 (3555–4875)	0.38
Urine output (ml)	1930 (1490–2393)	1703 (1296–2150)	*0.031*
Fluid balance (ml)	455 (-309–1153)	893 (-13–1538)	*0.035*
POD 2			
Fluid administered (ml)	4240 (3600–4856)	4075 (3500–4675)	0.43
Urine output (ml)	1880 (1400–2425)	1700 (1250–1970)	0.075
Fluid balance (ml)	720 (-353–1539)	728 (-263–1394)	0.92
POD 3			
Fluid administered (ml)	3800 (3200–4550)	3925 (3150–4425)	0.58
Urine output (ml)	2200 (1630–2913)	1960 (1350–2650)	0.125
Fluid balance (ml)	45 (-954–961)	370 (-1530–1175)	0.69
PODs 1–3 cumulative fluid balance (ml)	1603 (-545–2858)	1430 (-755–3495)	0.49

Intraoperative and postoperative fluid management, fluid balances, and medications. Data are presented as median (interquartile range, IQR) or number (percentage). *GDFT* goal-directed fluid therapy, *CFM* conventional fluid management, *POD* postoperative day

Postoperatively, the fluid balance in the GDFT group was lower on POD 1 than in the CFM group (455 ml vs. 893 ml, *p* = 0.035, Fig. 2). We found no differences in fluid management or fluid balances on PODs 2–4. Most patients in 2015 spent their first postoperative night in ICU (64.5%), whereas in 2017 fewer patients (24.6%) were admitted to intensive care. Examining the overall fluid management in ICU and on the surgical ward, POD 1 fluid administration was significantly lower in PD patients treated in ICU [median 3915 ml (IQR 3332–4533)] than in patients treated on the surgical ward [median 4100 ml (IQR 3750–4850), *p* = 0.037]. The median fluid balance remained lower in ICU patients on POD 1 [median 440 ml (IQR -272–1151)] than among surgical ward patients [median 863 ml (IQR -37.5–1593), *p* = 0.024]. We detected no differences in the fluid balance on PODs 2–4.

We found a significant difference in severe surgical complications requiring invasive intervention between the study groups (GDFT group, 19.4% vs. CFM group, 38.7%, *p* = 0.009). In addition, patients in the CFM group experienced clinically relevant pancreatic fistulas and post-pancreatectomy acute pancreatitis more often than patients in the GDFT group. Clavien–Dindo ≥ III complications occurred more often in the CFM group than in the GDFT group, although this difference remained insignificant. We found no differences in rates of pleural effusion, congestive heart failure, or pneumonias between groups. The length of hospital stay, however, was 9.0 days (IQR 7–14) in the GDFT group and 11.5 days (IQR 8–15) in the CFM group patients (*p* = 0.020). Table 3 summarizes all of the postoperative complications reported in the study groups.

**Table 3:** Postoperative complications

	GDFT group	CFM group	*p* value
Clavien–Dindo			0.16
0	10 (10.8%)	9 (12.0%)	
I	19 (20.4%)	7 (9.3%)	
II	39 (41.9%)	28 (37.3%)	
III	16 (17.2%)	24 (32.0%)	
IV	7 (7.5%)	6 (8.0%)	
V	2 (2.2%)	1 (1.3%)	
Clavien–Dindo 0–II	68 (73.1%)	44 (58.7%)	0.07
Clavien–Dindo III–V	25 (26.9%)	31 (41.3%)	
Severe surgical complications (invasive intervention)	18 (19.4%)	29 (38.7%)	*0.009*
Pancreatic fistula (grades B–C)	1 (1.1%)	8 (10.7%)	*0.011*
DGE (grades B–C)	20 (21.5%)	15 (20.0%)	0.25
PPH (grades B–C)	18 (19.4%)	21 (28.0%)	0.20
PPAP (grades B–C)	0 (0.0%)	4 (5.7%)	*0.042*
Chyle leak (grades B–C)	4 (4.4%)	2 (2.7%)	0.29
Abscesses	5 (5.4%)	7 (9.3%)	0.38
Congestive heart failure	11 (11.8%)	15 (20.0%)	0.20
Pleural effusion	5 (5.4%)	8 (10.7%)	0.25
Pneumonia	19 (20.4%)	13 (17.3%)	0.69
Reoperation	5 (5.4%)	7 (9.5%)	0.37
Readmission	19 (20.7%)	23 (31.1%)	0.15
LOS (days)	9.0 (7.0–14.0)	11.5 (8.0–15.0)	*0.020*

Postoperative complications. Data are presented as number (percentage) or median (IQR). *GDFT* goal-directed fluid therapy, *CFM* conventional fluid management, *DGE* delayed gastric emptying, *PPH* postpancreatectomy hemorrhage, *PPAP* post-pancreatectomy acute pancreatitis, *LOS* length of hospital stay

We more closely examined the perioperative fluid therapy in patients with specific complications. Patients with severe pancreatic fistulas (grades B–C) received more fluids on POD 1 than those without [median 5100 ml (IQR 4124–5500) vs. 4050 ml (3530–4622), *p* = 0.012]. They also had significantly higher fluid balances on POD 2 [median 1680 ml (IQR 1155–2145) vs. 540 ml (-350–1390), *p* = 0.005] and POD 3 [median 860 ml (IQR 738–1775) vs. 108 ml (-1240–1101), *p* = 0.004]. In addition, the cumulative fluid balance (PODs 1–3) was significantly higher in patients who later experienced pancreatic fistulas [median 4600 ml (IQR 1980–5418) vs. 1443 ml (-674–2905), *p* = 0.001]. Three patients with a severe pancreatic fistula also experienced post-pancreatectomy acute pancreatitis (33.3%). Furthermore, patients with pleural effusions had a higher cumulative fluid balance on PODs 1–3 than those without [median 2860 ml (IQR 136–4465) vs. 1443 ml (− 674–2922), *p* = 0.032].

Finally, we performed univariable and multivariable logistic regression analyses to identify risk factors for severe Clavien–Dindo complications (CD ≥ III) and for surgical complications requiring invasive intervention after PD (Table 4). These analyses indicated that a higher body mass index (BMI), a higher preoperative creatinine level, and falling in the CFM group all associated with severe complications. In addition, a pancreatic duct size ≤ 3 mm emerged as a risk factor only in the univariable analysis. In terms of severe surgical complications, a higher BMI and falling in the CFM group emerged as independent risk factors. In the subgroup analyses, the risk for severe Clavien–Dindo complications and for severe surgical complications were higher in the CFM group patients who received less than median intraoperative fluids (see Table 5).

**Table 4:** Univariable and multivariable analyses of severe postoperative complications

	Severe Clavien–Dindo complications						Severe surgical complications					
	Univariable			Multivariable			Univariable			Multivariable		
	*p* value	OR	95% CI	*p* value	OR	95% CI	*p* value	OR	95% CI	*p* value	OR	95% CI
Age	0.710	1.01	0.97–1.04	0.364	1.02	0.98–1.06	0.940	1.00	0.97–1.04	0.610	1.01	0.97–1.05
Gender (female)	0.380	0.75	0.39–1.43				0.808	0.92	0.47–1.81			
BMI (kg/m^2^)	< *0.001*	*1.21*	*1.10*–*1.33*	< *0.001*	*1.20*	*1.09*–*1.33*	*0.001*	*1.18*	*1.07*–*1.29*	*0.001*	*1.18*	*1.07*–*1.30*
Preoperative creatinine (µmol/l)	*0.016*	*1.02*	*1.00*–*1.04*	*0.035*	*1.02*	*1.00*–*1.04*	0.448	1.01	0.99–1.02	0.674	1.00	0.99–1.02
POD 1 creatinine (µmol/l)	*0.016*	*1.02*	*1.00*–*1.03*				0.240	1.01	0.99–1.02			
MPD ≤ 3 mm	*0.016*	*2.26*	*1.16*–*4.40*	0.173	1.70	0.79–3.65	0.066	1.91	0.96–3.81	0.350	1.46	0.66–3.20
Vascular resection	0.053	0.46	0.21–1.01				0.065	0.45	0.19–1.05			
Intraoperative fluids (ml/kg/h)	*0.019*	*0.88*	*0.82*–*0.98*				*0.016*	*0.88*	*0.79*–*0.98*			
Intraoperative albumin (g)	0.706	1.00	0.99–1.01	0.911	1.00	0.99–1.01	0.744	1.00	0.99–1.01	0.961	1.00	0.99–1.01
CFM group	*0.050*	*1.92*	*1.00*–*3.67*	*0.044*	*2.18*	*1.02*–*4.68*	*0.006*	*2.63*	*1.31*–*5.25*	*0.010*	*2.81*	*1.28*–*6.15*
GDFT group, intraoperative fluids above the median (ref.)		1						1				
GDFT group, intraoperative fluids below the median	0.197	2.00	0.70–5.73				0.077	2.80	0.89–8.77			
CFM group, intraoperative fluids above the median	0.733	0.81	0.20–3.24				0.658	1.39	0.33–5.85			
CFM group, intraoperative fluids below the median	*0.005*	*3.05*	*1.39*–*6.70*				*0.001*	*4.55*	*1.89*–*10.92*			
POD 1 in the ICU	0.665	0.86	0.45–1.67				0.386	1.37	0.68–2.76			
POD 1 balance	0.506	0.91	0.69–1.20				0.485	0.90	0.67–1.21			
POD 2 balance	0.414	1.11	0.86–1.43				0.338	1.14	0.87–1.48			
POD 3 balance	0.062	1.26	0.99–1.60				0.126	1.22	0.95–1.57			
Use of furosemide	0.052	2.14	0.99–4.60				0.075	2.11	0.93–4.78			

Risk factor analyses of severe postoperative complications (Clavien–Dindo ≥ 3) and severe surgical postoperative complications. *BMI* body mass index, *POD* postoperative day, *MPD* main pancreatic duct, *CFM* conventional fluid management, *GDFT* goal-directed fluid therapy, *ICU* intensive care unit

**Table 5:** Multivariable subgroup analyses of severe postoperative complications according to median intraoperative fluid volume

	Severe Clavien–Dindo complications			Severe surgical complications		
	*p* value	OR	95% CI	*p* value	OR	95% CI
Age	0.306	1.02	0.98–1.06	0.494	1.01	0.97–1.06
BMI (kg/m^2^)	*0.003*	*1.19*	*1.06–1.32*	*0.012*	*1.15*	*1.03–1.29*
Preoperative creatinine (µmol/l)	*0.048*	*1.02*	*1.00–1.04*	0.674	1.00	0.99–1.02
MPD ≤ 3 mm	0.159	1.74	0.81–3.75	0.321	1.49	0.68–3.30
Intraoperative albumin (g)	0.923	1.00	0.99–1.01	0.999	1.00	0.99–1.01
GDFT group, intraoperative fluids above the median (ref.)		1			1	
GDFT group, intraoperative fluids below the median	0.940	1.05	0.30–3.71	0.542	1.50	0.41–5.57
CFM group, intraoperative fluids above the median	0.891	1.11	0.25–4.83	0.536	1.61	0.36–7.20
CFM group, intraoperative fluids below the median	*0.041*	*2.58*	*1.04–6.42*	*0.007*	*3.75*	*1.44–9.77*

Subgroup analyses of severe postoperative complications (Clavien–Dindo ≥ 3) and severe surgical postoperative complications according to intraoperative median fluid volume. Fluid management groups and intraoperative fluid volume were arranged to a single variable as they are correlated and related to each other. *BMI* body mass index, *POD* postoperative day, *MPD* main pancreatic duct, *CFM* conventional fluid management, *GDFT* goal-directed fluid therapy, *ICU* intensive care unit

## Discussion

We found that goal-directed fluid therapy (GDFT) led to a higher intraoperative fluid volume than conventional fluid management (CFM), a finding contrary to our original hypothesis. In addition, the fluid balance on POD 1 was lower in the GDFT group. Patients in the GDFT group experienced fewer severe surgical complications, pancreatic fistulas**,** and post-pancreatectomy acute pancreatitis, and the length of a hospital stay was shorter than among patients in the CFM group.

Evidence from previous studies indicated that GDFT leads to a lower intraoperative fluid administration,^31^ lower intraoperative fluid balance, and better urine output than a liberal fluid management.^32–34^ Our results contradict previous studies concerning fluid balance and fluid administration. However, the conventional fluid management employed in our study cannot be defined as a liberal fluid therapy, and thus our study design was somewhat distinct from those earlier studies. Moreover, our GDFT protocol was slightly different: the crystalloid:albumin ratio was 3:1, such that fluid management with crystalloids was more generous than that reported by other researchers. In addition, inotropes were not a part of the algorithm even if norepinephrine was required, while other studies reported that inotropes formed one part of the GDFT protocol.^32,34^ Nevertheless, the GDFT group in our study only received intraoperative fluids when the patient hemodynamics indicated a need for fluid administration. Thus, fluid management was controlled and excessive fluids were avoided. Furthermore, intraoperative monitoring was more precise in the GDFT group due to use of the EV1000 device.

The median intraoperative fluid administration in the GDFT group was 4.8 L falling to 3.3 L in the CFM group. In the large RELIEF study, which explored the fluid management of patients undergoing major abdominal surgery, the median liberal fluid administration reached 6.1 L (IQR 5.0–7.4), falling to 3.7 L (IQR 2.9–4.9) in the restrictive group. Given that study, the fluid administration in the GDFT group in our study approaches a classic definition of liberal fluid administration, whereas the CFM group controversially remains close to restrictive.^35^ A liberal or restrictive approach was not the aim in either group. A large retrospective study of noncardiac surgical patients demonstrated that moderate intraoperative fluid therapy yields the best postoperative results: a too-restrictive or a too-liberal fluid therapeutic approach increases the complication rates and the length of a hospital stay. In that study, a moderate fluid therapy was 6–7 ml/kg/h, although they included all noncardiac surgical procedures in the study hospitals.^36^ In comparison, PD is a major procedure with extensive surgical wounds, and significant evaporation during surgery is more common compared to smaller surgical procedures. Thus, a moderate fluid therapy in PD surgery may include a higher volume of infusions.

The only difference between our study groups with regard to postoperative fluid management was that GDFT patients had a lower fluid balance on POD 1 and a higher urine output than the CFM group. The ward in which patients spent their first postoperative night after surgery may have affected outcomes. The hospital practice patterns concerning the postoperative care changed between cohorts, whereby patients in 2017 were less frequently admitted postoperatively to ICU than were patients in 2015. Compared to patients in a regular surgical ward, POD 1 fluid administration and fluid balances were lower among ICU patients, possibly because of the use of norepinephrine administered to maintain a sufficient arterial blood pressure and the use of diuretics to improve urine output. However, the ward in which the patient spent the first postoperative night had no statistically significant impact on the occurrence of severe postoperative complications in the logistic regression analyses.

Postoperative fluid therapy and fluid balance may impact patient outcomes. Previous studies indicated that the entire perioperative fluid management carries more significance than the intraoperative period alone. For instance, Behman showed that a higher postoperative fluid balance until POD 2 following PD associated with an increased incidence of complications, more frequent postoperative ICU admission, and a longer hospital stay.^37^ In another study, higher fluid balances 48 and 72 h after surgery emerged as risk factors for a higher morbidity and a longer hospital stay.^38^ Yet, these studies were retrospective and a meta-analysis in 2018 provided no conclusions regarding the association between postoperative fluid administration and complication rates given the scarcity of such studies.^24^ More prospective studies are needed to verify such an association. Ultimately, in our univariable analysis, postoperative fluid management had no statistically significant influence on postoperative outcomes.

The overall postoperative complication rate was rather high in the CFM group, whereby 41.3% of patients experienced a severe complication (Clavien–Dindo ≥ III) compared with 26.9% in the GDFT group. This latter finding mirrors the complication rate reported in previous studies.^3^ That said, each complication increases costs and prolongs the hospital stay after PD.^39^ In our study, the GDFT group recovered more quickly and the CFM group had a significantly longer hospital stay, which presumably affected the costs of the postoperative period.

One major difference in complication rates was seen in clinically relevant pancreatic fistulas (grades B–C). Only one severe pancreatic fistula occurred in the GDFT group, whereas in the CFM group over 10% of patients experienced that specific complication. While the surgeons were the same during both study periods, this difference in complications is notable. A high net 72-h fluid balance has been singled out as an independent predictor of postoperative pancreatic fistulas following PD,^40^ and pancreatic fistula patients had significantly higher net fluid balances on PODs 2–3. Despite the small number of patients with pancreatic fistulas, our study thus supports those previous findings.

Our multivariable findings confirm the result that PD patients benefit from a goal-directed approach to fluid management. A restrictive approach without monitoring the response to the administration of fluids proved to be an inferior choice. A previous meta-analysis reported that GDFT leads to fewer complications in abdominal surgery patients^41^; in our study, the benefit was similar for patients undergoing PD. A large Japanese registry study indicated that risk factors for severe complications following PD include, for example, male gender, age, a high BMI, ASA grade ≥ 3, smoking, comorbidities, a high preoperative creatinine, low plasma albumin levels, and combined vascular resection.^42^ In our study, the risk for severe complications was increased with a high BMI and a high preoperative creatinine level, thereby partly agreeing with that Japanese study.

Our study has some limitations given its retrospective design. We were obliged to rely on patient records and, as such, variables such as fluid administration postoperatively on the day of surgery were impossible to define precisely. Although the intraoperative fluid management was performed according to a GDFT protocol in 2015, the other modalities for anesthesia were not standardized. The postoperative treatment of patients followed the same ERAS protocol in both cohorts; but, given the retrospective study design, slight individual differences may have occurred. Furthermore, the department performing pancreatic surgery relocated during the follow-up period: the GDFT group was treated in a smaller hospital specialized in abdominal surgery, and the CFM group received treatment in a larger unit with expertise in pancreatic surgery, alongside a wide spectrum of different surgical patients and a larger staff. The surgeons were the same in both cohorts and the experienced staff followed the patients, but it remains unclear whether some treatment conventions varied when moving to the larger unit. Due to the retrospective design of this study, we can only provide associations. In addition, the effect of different pancreatic textures could not be analyzed in the patient groups, because no description of the texture was noted in the records for a majority of the patients. Patients with a soft pancreatic texture could be interesting to analyze as a separate group.

## Conclusions

PD patients benefit from targeted and controlled fluid administration according to hemodynamic parameters. Accurate monitoring and reacting promptly to changes improves patient outcomes. Treatment in a high-dependency unit during the first postoperative night may improve monitoring, and reduce severe complications and the total length of a hospital stay. Perioperative fluid management should be considered in its entirety. Prospective studies are needed to understand the relationship between postoperative fluid management and complications.
